# The Binding of Triclosan to SmeT, the Repressor of the Multidrug Efflux Pump SmeDEF, Induces Antibiotic Resistance in *Stenotrophomonas maltophilia*

**DOI:** 10.1371/journal.ppat.1002103

**Published:** 2011-06-30

**Authors:** Alvaro Hernández, Federico M. Ruiz, Antonio Romero, José L. Martínez

**Affiliations:** 1 Centro Nacional del Biotecnología, CSIC, Cantoblanco, Madrid, Spain; 2 Centro de Investigaciones Biológicas, CSIC, Madrid, Spain; University of California San Diego, United States of America

## Abstract

The wide utilization of biocides poses a concern on the impact of these compounds on natural bacterial populations. Furthermore, it has been demonstrated that biocides can select, at least in laboratory experiments, antibiotic resistant bacteria. This situation has raised concerns, not just on scientists and clinicians, but also on regulatory agencies, which are demanding studies on the impact that the utilization of biocides may have on the development on resistance and consequently on the treatment of infectious diseases and on human health. In the present article, we explored the possibility that the widely used biocide triclosan might induce antibiotic resistance using as a model the opportunistic pathogen *Stenotrophomonas maltophilia*. Biochemical, functional and structural studies were performed, focusing on SmeDEF, the most relevant antibiotic- and triclosan-removing multidrug efflux pump of *S. maltophilia*. Expression of *smeDEF* is regulated by the repressor SmeT. Triclosan released SmeT from its operator and induces the expression of *smeDEF*, thus reducing the susceptibility of *S. maltophilia* to antibiotics in the presence of the biocide. The structure of SmeT bound to triclosan is described. Two molecules of triclosan were found to bind to one subunit of the SmeT homodimer. The binding of the biocide stabilizes the N terminal domain of both subunits in a conformation unable to bind DNA. To our knowledge this is the first crystal structure obtained for a transcriptional regulator bound to triclosan. This work provides the molecular basis for understanding the mechanisms allowing the induction of phenotypic resistance to antibiotics by triclosan.

## Author Summary

The wide utilization of biocides for different purposes, including toothpastes, soaps, house-hold compounds surfaces' disinfectants and even their use as additives of different materials (from textiles to concrete used in germ-free buildings) to avoid their colonization by microorganisms, poses a concern on the impact of these compounds on natural bacterial populations. Furthermore, it has been demonstrated that such biocides can select, at least in laboratory experiments, bacteria resistant to antibiotics. This situation has raised concerns on the impact that the utilization of biocides may have on the development on resistance and consequently on the treatment of infectious diseases. In the present article we study whether biocides can induce phenotypic resistance to antibiotics, a process that would be barely detectable unless purposely searched out. In the article, we present functional, biochemical and structural data showing that the widely used biocide triclosan induces antibiotic resistance, mediated by the binding of the biocide to SmeT, the transcriptional regulator of the expression of the *Stenotrophomonas maltophilia* multidrug efflux pump SmeDEF, which can extrude an ample range of antibiotics. Our study provides an unambiguous link between the presence of this biocide and the increased efflux of antibiotics by the opportunistic pathogen *S. maltophilia*.

## Introduction

The widespread use of biocides in toothpastes, soaps, household cleaning agents, surface disinfectants and as additives in different materials (from textiles to the concrete used in germ-free buildings) etc., all with the aim of preventing microbial colonization [1]–[5], could have an undesired impact on natural bacterial populations [1], [6]–[8]. Biocides have been associated with the *in vitro* selection of bacterial mutants showing reduced susceptibility to antibiotics (cross-resistance) without the need for any antibiotic-selective pressure [9]–[12], although whether this occurs in the wild is less clear. Triclosan is one of the most widely used biocides [13]. Using different models it has been shown that resistance to triclosan can be conferred by the expression of multidrug (MDR) efflux pumps capable of expelling antibiotics [9], [11], [14], [15]. Mutants overexpressing MDR efflux pumps are easily obtained under antibiotic selective pressure [16]–[18]. It has also been shown that triclosan can select for mutants that constitutively overproduce such pumps and which are thus less susceptible to antibiotics [9], [11], [14], [15]. The constitutive overexpression of MDR efflux pumps is very often due to mutations in the local transcriptional regulators that control pumps expression or, in a few cases, to mutations in their operator DNA sequences [19]–[21].

The expression of chromosomally-encoded MDR efflux pumps is tightly controlled by specific transcriptional regulators (usually repressors). Under normal growing conditions in the laboratory, MDR efflux pumps are expressed at a very low level (if they are expressed at all) [9], [11], [14], [15]. However, their expression can be activated by the binding of effectors to their repressors and the consequent inhibition of the binding of such repressors to their operators [22]–[27]. Although most work on bacterial efflux pumps has focused on their impact on antibiotic resistance, antibiotics are not always the natural inducers of their expression [20]. In fact, in spite of the broad range of substrates that efflux pumps can expel, only a narrow group of ligands can act as effectors capable of triggering the transcription of the operons encoding these pumps.

The present work explores whether the biocide triclosan can activate the expression of MDR efflux pumps. Previous work has shown that triclosan selects mutants that overproduce the *Stenotrophomonas maltophilia* MDR efflux pump SmeDEF [11]. This efflux pump belongs to the resistance-nodulation-cell division family and is a tripartite efflux pump formed by an inner membrane protein, which is the transporter itself (SmeE), an outer membrane protein (SmeF) and a membrane fusion protein (SmeD). *S. maltophilia* is often isolated from the rhizosphere and from water sources [28], [29]. Besides this environmental origin, this bacterial species is an opportunistic pathogen, which presents low susceptibility to several antibiotics [30], [31], and is involved in different types of infections with a considerable mortality rate [32]. Infections by *S. maltophilia* include bacteremia [33], endocarditis [34], infections in patients with cancer [35] and respiratory tract infections, including those suffered by cystic fibrosis patients [36]–[38] among others. The genome of this bacterial pathogen harbors a large number of antibiotic resistance determinants [39], [40], including antibiotic inactivating enzymes [41]–[43], a *qnr* determinant [44]–[46] and different MDR efflux pumps, like SmeABC, SmeDEF, SmeJKL and SmeYZ, being SmeDEF the most important MDR efflux pump known to confer antibiotic resistance in *S. maltophilia* [47]–[52]. The expression of SmeDEF is regulated by SmeT, a transcriptional repressor encoded upstream of *smeDEF* in the complementary DNA strand [53], [54]. SmeT belongs to the TetR family of transcriptional repressors. The members of this family show a characteristic helix-turn-helix DNA-binding motif at their N-terminal end and a C-terminal region involved in both dimerization and effector binding [55]. The structural analysis of SmeT has revealed this repressor to have close similarities to TetR, QacR and TtgR [53] and to a lesser extent with CprB [56], EthR [57], CmeR [58], AcrR [59], ActR [60], IcaR [61], members of the TetR family of repressors. However, unlike them, SmeT has extensions at its termini that might modulate its interaction with DNA as well as the nature and size of the effector-binding pocket (when empty, this pocket is the smallest of all those of the TetR family members). SmeT binds to a 28 bp-long pseudopalindromic region in the promoter regions of *smeDEF* and *smeT* with a Km (app.), calculated from the data presented in [53] in the range of 1 µM, which is similar to that found for the TetR regulator TtgR [62]. The binding of SmeT to its operator region simultaneously represses *smeDEF* and *smeT* transcription by the steric interference of RNA polymerase binding to DNA [54]. Constitutive overexpression of *smeDEF* occurs in mutants selected by triclosan or antibiotics, and these show changes in SmeT that preclude the binding of the repressor to its operator [48], [51]. The possibility of the binding of effectors to SmeT inducing *smeDEF* expression has been suggested [53], but never demonstrated.

SmeDEF has a wide range of substrates that includes antibiotics, solvents, biocides and dyes [47], [49]. However, no information is available on the inducers of this efflux pump. To ascertain whether the biocide triclosan, which is a substrate of SmeDEF, might also activate its expression, a number of functional and structural analyses were performed. The data collected support the idea that triclosan can induce the expression of *smeDEF* and consequently reduce the susceptibility of *S. maltophilia* to antibiotics. This induction is due to the binding of triclosan to the pump repressor SmeT, which impedes its binding to its operator region, and triggers the expression of the most important MDR system in *S. maltophilia*, SmeDEF. The X-ray crystal structure of the SmeT-triclosan complex indicates that the biocide stabilizes the protein structure in a conformation unable to bind DNA. To our knowledge, this crystal structure is the first structural evidence of the ability of triclosan to act as an effector via its binding to a transcriptional regulator. Given that this regulator (SmeT) mediates the susceptibility of *S. maltophilia* to antibiotics by repressing *smeDEF* expression, the present results provide information that aids our understanding of the molecular basis of biocide-induced antibiotic resistance.

## Results

### Triclosan modifies the fluorescence spectra of SmeT

**Figure 1:**
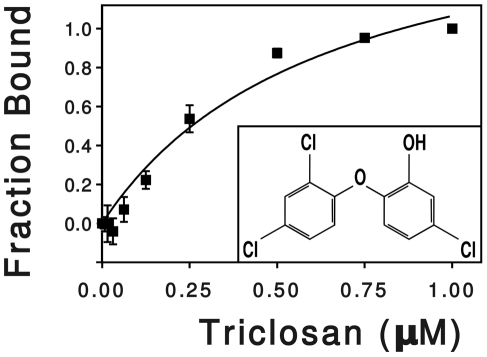
Effect of triclosan concentration on the fluorescence of SmeT. SmeT was treated with increasing concentrations of triclosan at room temperature. The change in fluorescence, relative to the untreated sample, was plotted against triclosan concentration. The hyperbola shows the best fit of the data. The chemical formula of triclosan (5-chloro-2-(2,4-dichlorophenoxy) phenol) is shown into the box.

To measure the binding kinetics of triclosan to SmeT, we determined the changes of fluorescence of SmeT in the presence of triclosan. This method has been previously used for analyzing the interactions of triclosan with the enoyl-acyl carrier protein reductase [63] and is a good alternative to isothermal titration calorimetry for molecules with low solubility in water as triclosan. The triclosan addition to SmeT samples resulted in a concentration-dependent quenching of the intrinsic protein fluorescence. The fluorescence variations, relative to the untreated samples, were best fitted by a single hyperbola, assuming an stoichiometry of two molecules of triclosan per SmeT dimer and yielding an apparent K_d_ value of 0.63±0.15 µM (Figure 1). Values for the K_d_ in the low micro molar range have been described for the binding of drugs to different transcriptional regulators of the TetR family [24], [64]. These results confirmed that in the solution state triclosan interacts with SmeT at concentrations similar to those described for known effectors of other members of the TetR family of transcriptional regulators.

### Triclosan releases SmeT from its operator DNA

**Figure 2:**
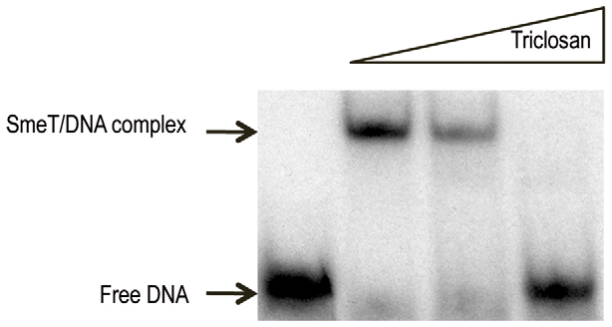
Triclosan breaks the SmeT-DNA complex. The γ-[32P] labeled 30 bp operator DNA (2 nM, 10000 cpm) of SmeT was incubated with 0.2 µM SmeT for 20 min at room temperature (lane 1). Subsequently, increasing concentrations (0.1 mM and 0.2 mM) of triclosan were added and the mixture further incubated at room temperature for 15 min more. Retarded complexes were separated in a 6% non-denaturing polyacrylamide gel. The positions of free DNA and the retarded SmeT-DNA complex are indicated with arrows.

It has been shown that the binding of other members of the TetR family to their cognate DNA operators is modulated in response to effectors such as antibiotics, detergents or plant exudates [55]. To determine whether triclosan is able to induce a conformational change in SmeT and thus modify its DNA binding properties, EMSA was performed with SmeT and a 30-bp DNA fragment containing its operator either in the presence or absence of the biocide. *In vivo*, SmeT is usually bound to its operator thus repressing transcription of *smeDEF*. However, the entrance of an effector into its binding pocket might release the effector-SmeT complex from the DNA. To mimic this situation, triclosan was added to preformed SmeT-DNA complexes. As shown in Figure 2, the addition of triclosan to the DNA-SmeT complex resulted in the loss of the retarded band, indicating the separation of the components. The addition to the preformed complex of ciprofloxacin, which is a substrate of SmeDEF [49], did not release SmeT from its operator (not shown). These results suggest that the structural changes suffered by SmeT upon triclosan binding render it unable to bind to its cognate operator DNA.

### Triclosan induces the transcription of smeDEF

**Figure 3:**
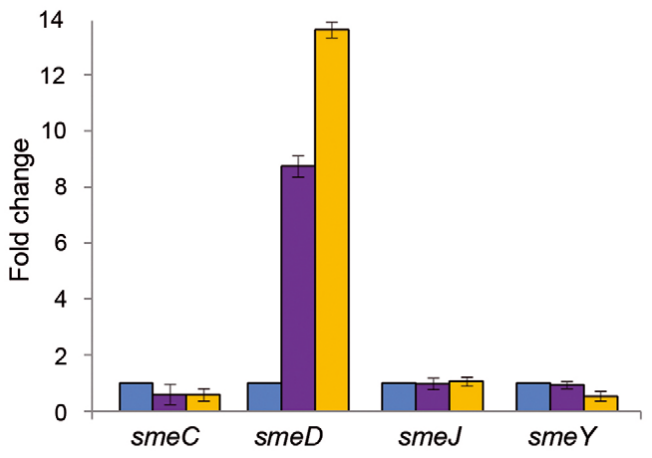
Triclosan increases the mRNA levels of *smeD*. The amount of *smeC*, *smeD*, *smeJ* and *smeY* mRNAs in the presence of triclosan (purple columns) was measured by real time RT-PCR and the fold changes estimated with respect to the value determined for the wild type strain grown in the absence of the biocide (blue columns). As shown, the expression of *smeD* was the only one induced by triclosan. D457R (yellow columns) is a mutant strain in which *smeDEF* is fully de-repressed due to a mutation that inactivates SmeT. This mutation did not affect the expression of the other tested MDR efflux pumps.

Since the addition of triclosan precludes the *in vitro* binding of SmeT to its cognate operator, *smeDEF* expression ought to be induced by this biocide. To ascertain whether our *in vitro* data match the physiological *in vivo* response, the levels of the mRNA from the *smeD* gene in the presence and absence of the biocide were measured by real time RT-PCR (Figure 3). The expression of *smeD* was also measured in *S. maltophilia* D457R. This strain is a natural mutant, derived from the wild-type D457, which has been selected in the presence of antibiotics [50]. The multidrug resistant strain D457R harbors an inactive allele of SmeT as the consequence of a Leu166Gln change [51], [54]. Because of this, the strain D457R constitutively expresses high levels of *smeDEF* [49] and it is thus a good control for measuring the level of expression of *smeDEF* under non-repressing conditions. As shown in Figure 3, triclosan increased 8.7-fold the expression levels of *smeD* compared to the levels observed for cells growing without the biocide. In comparison, *S. maltophilia* D457R, in which *smeDEF* transcription is fully de-repressed, showed a 13.7-fold increase for *smeD* mRNA. These results indicate that triclosan de-represses the transcription of *smeDEF* in agreement with the data obtained with the EMSA assays described above.

The genome of *S. maltophilia* harbors genes encoding several putative MDR efflux pumps [40]. Among them, SmeABC, SmeJKL and SmeYZ are known to be involved in antibiotic resistance in *S. maltophilia* [40], [65], [66]. To determine whether the effect of triclosan was specific for *smeDEF* or whether other MDR pumps are induced by the biocide, the expression of *smeC*, *smeJ* and *smeY* was examined. As shown in Figure 3, none of these MDR pumps were induced by triclosan, indicating that the effect of this biocide is specific for *smeDEF*.

### Triclosan reduces the susceptibility of *S. maltophilia* to quinolones

Since triclosan induced the expression of *smeDEF*, it was predicted that the susceptibility of *S. maltophilia* to antibiotics would be lower in the presence of the biocide. To test this, Etest assays were performed with the biocide and ciprofloxacin. Ciprofloxacin was chosen because the constant over-production of SmeDEF in the strain *S. maltophilia* D457R, which harbors a defective SmeT repressor, results in an 8-fold increase in the MIC value for this quinolone [48], and because ciprofloxacin does not induce *smeDEF* expression (AH, unpublished results).

**Figure 4:**
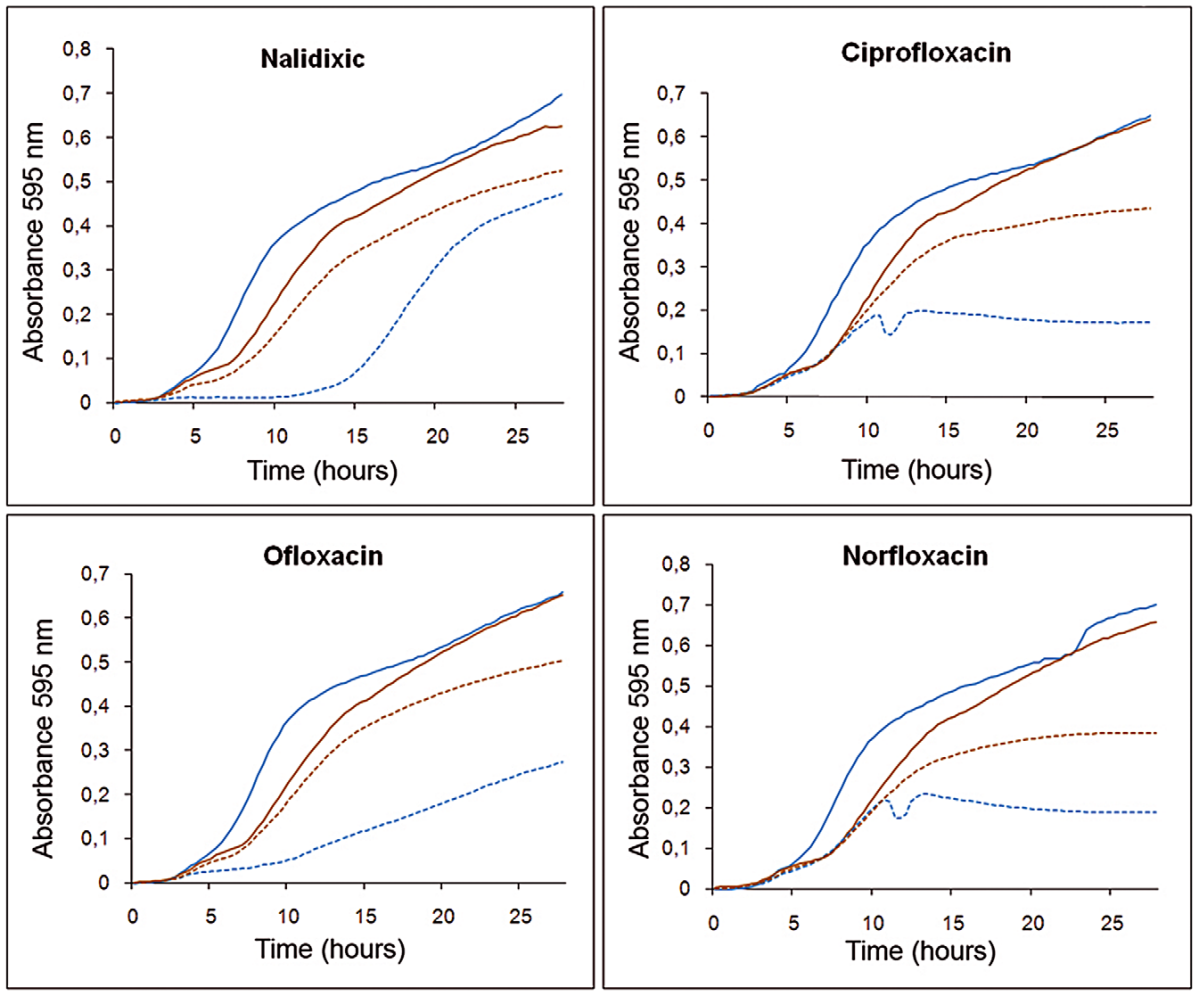
Effect of triclosan and quinolones alone or in combination on the growth of *S. maltophilia*. Bacterial growth in presence of 3 µg/ml triclosan (solid brown lines) was slightly impaired compared with that in absence of the biocide (solid blue lines). However, in the presence of an antibiotic concentration that precluded bacterial growth this effect was reverted, and bacteria growing with triclosan and antibiotic (dotted brown lines) had an ameliorated growth compared to those growing in presence of the antibiotics but without the biocide (dotted blue lines).

For these assays, a square of dried Whatman paper previously soaked with triclosan was placed just below the point of the Etest strip corresponding to the minimal inhibitory concentration (MIC) of ciprofloxacin, and the MICs in the presence or in the absence of the biocide were determined. A 2.5-fold increase in ciprofloxacin MIC was observed in the presence of triclosan (from 0.75 µg/ml to 2 µg/ml), indicating that the biocide induced resistance to antibiotics, although the increase in MIC was lower than that observed in the *S. maltophilia* D457R mutant, which constitutively expresses *smeDEF* at high level [48]. To further confirm that triclosan transiently reduces the susceptibility of *S. maltophilia* to quinolones, growth curves were plotted for *S. maltophilia* cultures with or without triclosan in the presence or absence of sub-MIC concentrations of these antibiotics. As shown in Figure 4, at the tested concentrations the presence of the biocide alone slightly slowed the growth of *S. maltophilia*. However, when bacterial growth was inhibited by adding the antibiotics, the presence of the biocide favored bacterial growth, antagonizing the inhibitory effect of the quinolones. This indicates that the biocide exerts a dual effect on bacterial viability and thus on the susceptibility of *S. maltophilia* to antibiotics (Figure 4). Triclosan inhibits bacterial growth but simultaneously induces the expression of drug-detoxification elements. This mixed effect might be the cause of the moderate increase in MIC values observed in the presence of triclosan, in spite of highly increased *smeD* expression.

### Binding of triclosan to SmeT: overview of the structure of the complex

**Figure 5:**
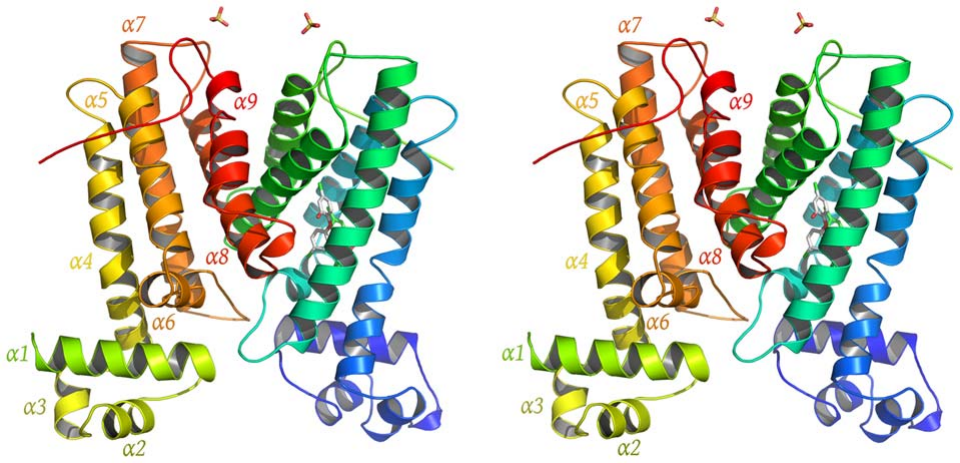
Overview of SmeT-triclosan structure. Stereo cartoon representation of the SmeT-Triclosan complex structure, with triclosan in stick representation. Helices are shown in different colors; for clarity they are labeled only in monomer B.

To gain more insight into the structural basis of the induced expression of SmeDEF by triclosan, SmeT was co-crystallized with this biocide and the structure solved by X-ray diffraction. The structure of the SmeT-triclosan complex involves one homodimer in the asymmetric unit, as seen for other TetR family members [55]. Each SmeT polypeptide chain is composed of 9 α-helices (α1–α9) divided into two structurally distinct domains (Figure 5). The smaller N-terminal domain, composed of the first 3 helices (α1: residues 14–27, α2: residues 24–41, α3: residues 45–49) and the beginning of the fourth, mediates DNA binding through the N-terminal helices α2 and α3, which are almost perpendicular to each other and constitute the DNA-binding helix-turn-helix motif. The larger C-terminal domain, which is mainly involved in ligand binding and dimerization, is composed of helices 4 to 9 (α4: residues 54–76, α5: residues 85–102, α6: residues 104–114, α7: residues 127–149, α8: residues 159–179 and α9: residues 186–201). The dimerization surface, which is mostly formed by α8 and α9 helices, has a hydrophobic character despite the highly negatively charged solvent-exposed surface of the C-terminal domain. This interface involves 48 residues of chain A and 46 residues of chain B, an area of 1718 Å^2^ and 1796 Å^2^ respectively. The hydrophobic interactions are complemented by a network of at least eight hydrogen bonds and two salt bridges between Arg134 (chain A) and Glu180 (chain B) and between Arg164 and Asp189 of both monomers. The monomers are almost identical, with a root mean square deviation (rmsd) of 0.708 Å for all atoms (0.680 Å for Cα atoms). The biggest difference between monomers is found at the N terminal domain, with a rmsd value of 0.712 Å compared to 0.611 Å for the C-terminus.

**Figure 6:**
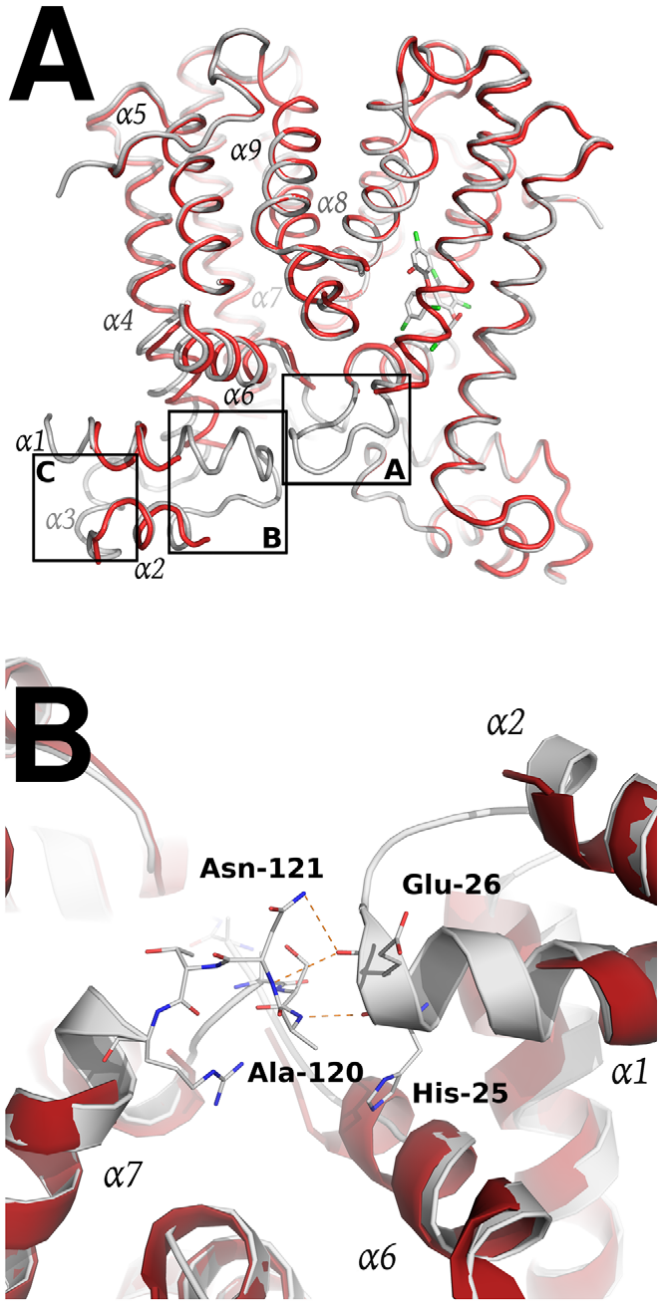
Structural comparison between the apo SmeT (in red) and the SmeT-Triclosan complex (in grey) structures. (A) The binding of triclosan (shown in stick representation) induces the stabilization of the loop connecting helices α6 and α7 in the subunit A (box A). This loop stabilizes through H-bonds the loop connecting helix α1 and α2 (box B), making more stable the N terminal domain of the second subunit, in particular the helix α3 (box C). (B) Close-up view of the SmeT dimer interface in the N-terminal domain showing the interactions between the loop connecting helices a6 and α7 and the loop connecting helices α1 and α2, a region poorly defined in the SmeT structure.

The SmeT-triclosan structure revealed several structural differences with respect to the apo SmeT structure [53] that shed light into the role of the biocide at the binding pocket and its subsequent stabilization of the protein folding specially at disordered regions that could not be modeled in the apo SmeT structure (Figure 6a). Residues 10–12, 21–34 and 45–55 that appeared disordered in apo SmeT become well ordered upon triclosan binding. Therefore, a new interface area between the loop connecting helices α6 and α7 (residues Ser116 – Arg123) and the top of helix α1 (residues His25 – Gly28) and the beginning of the loop connecting α1 and α2 (residues Val29 – Thr33) could be seen as a result of this ordering (Figure 6b). In this area, poorly defined in the SmeT structure [53], hydrogen-bonds formed between Ala120 and Asn121 of one monomer and His25 and Glu26 of the other, respectively, help to stabilize this region.

### Triclosan binding site

**Figure 7:**
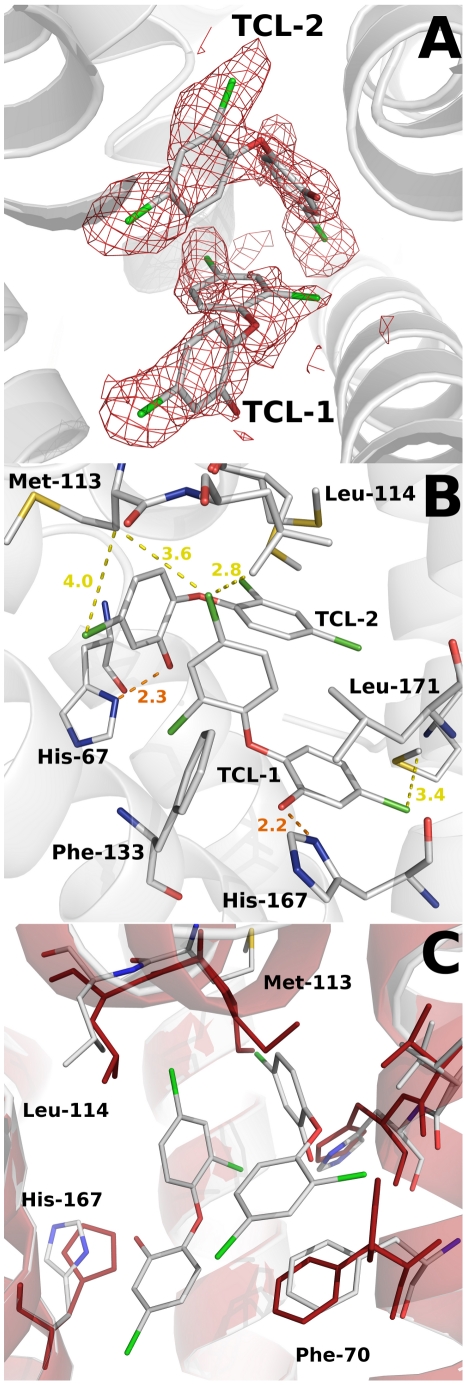
Structural details of the triclosan binding site. (A) The Fo-Fc omit electron density map contoured at 2.4 σ around the triclosan molecules is shown in red. (B) Key interactions (H-bonds in orange) between the triclosan molecules and the binding pocket residues of SmeT (distances less than 4 Å). The most important residues in the formation of the binding pocket are shown and labeled. (C) The binding of two molecules of triclosan displaces residues His-167, Leu-114 and Met-113. These last two residues belong to helix a6, which interacts trough H bonds with helix a1, part of the DNA binding domain of SmeT.

A closer inspection of the electron density maps in the C-terminal domain showed a clear density in one of the subunits (A) (Figure 7a). This density was readily interpretable and allows the unambiguous placement of two molecules of the biocide (in agreement with the stoichiometry determined by fluorescence measurements) and the subsequent refinement. This is the first structural evidence showing a transcriptional regulator in complex with triclosan. However, no equivalent dense area was seen in subunit B. The volume of the triangle-shaped ligand binding cavity is 1030 Å^3^, which represents a notable increase with respect to the volume calculated for the apo SmeT structure (630 Å^3^), but similar to the calculated values for other TetR proteins.

One of the triclosan (TCL1) molecules binds to the bottom of the ligand binding site, in close contact with helices α6, α7 and α8. The phenolic hydroxyl group forms a strong hydrogen bond with the Nδ atom of His167 (placed at 2.2 Å), anchoring this molecule almost parallel to helix α8. In the apo SmeT structure this residue is placed at an alternative conformation into the empty binding site, with the Cδ and Nε atoms pointing to the interior of the cavity. The position and orientation of the triclosan molecule is further stabilized through its 2,4-dichlorophenoxy ring via two edge-to-face aromatic ring interactions, on one side with Phe133 (3.9 Å) and on the other with the 2,4-dichlorophenoxy ring of the second triclosan molecule (TCL2) (4.1 Å). In this scenario the chlorine atoms engage stabilizing interactions with hydrophobic residues at the binding site. Thus, the 5-Cl atom makes contact with the side chains of Val170 (3.5 Å), Leu166 (4.1 Å), Met140 (3.3 Å) and Met93 (3.5 Å). The 2-Cl atom faces Gly132 (3.2 Å), the Cε atom of His67 (3.3 Å), and the phenol ring of TCL2 (4.4 Å). The 4-Cl atom is surrounded by the side chains of Met110 (3.4 Å), Leu114 (3.6 Å) and Met113 (3.6 Å). This atom, together with the phenolic ring of TCL2, displaces these last two residues to expand the active site cavity (see above) and maybe to accommodate a second triclosan molecule (Figure 7b). The hydroxyl group of the phenolic ring of TCL2 forms an H-bond with the Nδ atom of His67 (2.3 Å). The 2,4-dichlorophenoxy ring of TCL2 stacks against the phenol ring of Phe70 (3.4 Å). This residue was modeled in double conformation in the apo SmeT structure but only the so-called open conformation is seen in the structure of the SmeT-triclosan complex (Figure 7c). The density for this residue in subunit B is weaker than in subunit A and a single conformation of the side chain was modeled with an occupancy of 50%. None of the triclosan molecules interacts with residues from subunit B.

## Discussion

In recent years, the possibility that widely-used biocides might co-select for antibiotic resistance has been suggested to pose a potential risk to the successful treatment of infectious diseases [1], [6]–[8]. Although presently there is no clear evidence of the selection of antibiotic-resistant mutants by biocides in the wild, risk-assessment studies are required since *in vitro* experiments have shown that exposure of bacterial populations to certain biocides, such as triclosan, indeed leads to selection for mutants with reduced susceptibility to antibiotics [11], [14], [67], [68]. On most occasions on which the molecular basis of this resistance has been explored, it has been acquired as a consequence of the stable de-repression of MDR efflux pumps [9], [11], [14], [15].

**Figure 8:**
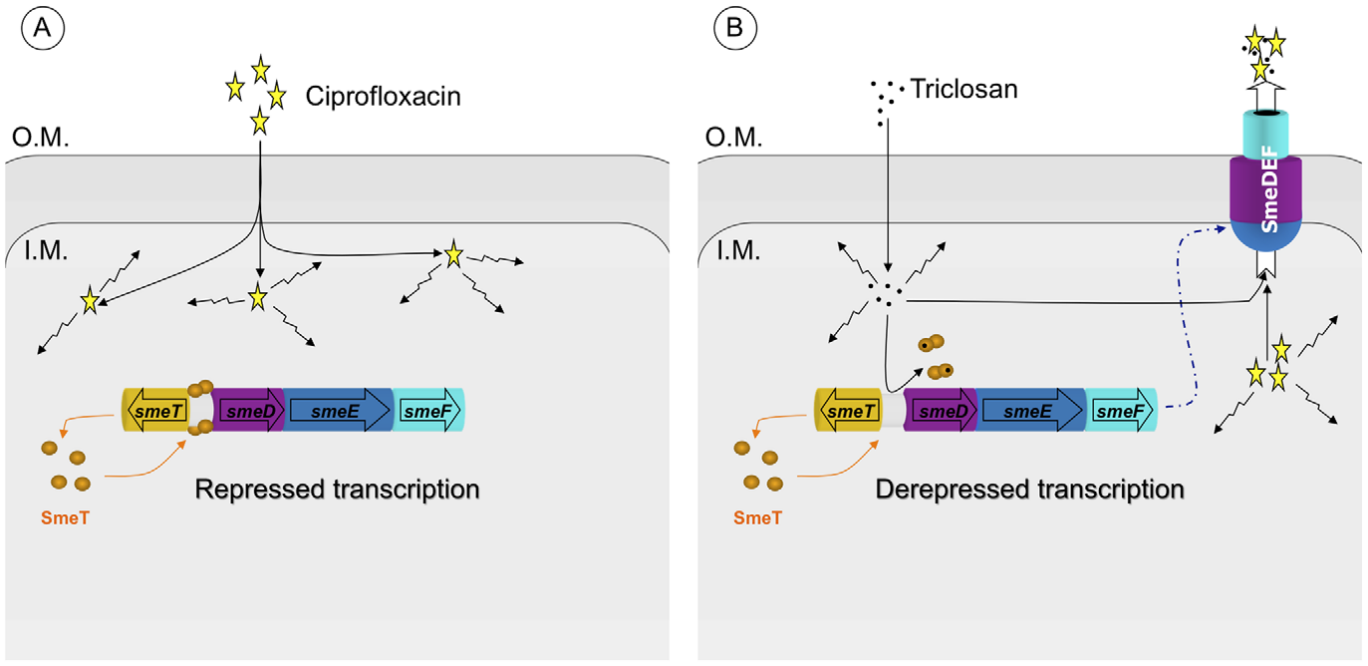
Effect of triclosan on the susceptibility to antibiotics of *S. maltophilia*. Panel A: In the basal state, SmeT (orange circles) is bound to its cognate operator DNA repressing the transcription of *smeDEF*. The fact that damage is caused by ciprofloxacin (yellow stars) inside the bacteria is represented by crinkly arrows. Panel B: The entrance of triclosan (black spots) causes bacterial damage and, simultaneously, the binding of the biocide to SmeT releases the protein from its operator allowing high-level transcription of *smeDEF*. Once the pump is expressed, its substrates - triclosan and ciprofloxacin - are pumped out of the bacteria. However the toxic effect of triclosan prevents the cells reaching the level of resistance observed in a mutant defective in SmeT (see text for more details).

Since the expression of chromosomally-encoded MDR efflux pumps is usually strongly down-regulated by local repressors [19], and their expression can be triggered by specific effectors [27], the present work explores the possibility that triclosan, a known substrate of the *S. maltophilia* MDR efflux pump SmeDEF [11], might induce the expression of this antibiotic resistance determinant and thus render an inducible phenotype of antibiotic resistance that would be barely detectable unless searched for. Triclosan-induced phenotypic antibiotic resistance [69] is a possibility since previous work by our group has shown that the exposure of *S. maltophilia* to triclosan selects for antibiotic resistant mutants that overexpress SmeDEF [11]. The present results show that triclosan binds the repressor of *smeDEF* transcription SmeT, and that this binding induces a conformational change in SmeT, which is released from its DNA operator. Although additional crystal structure of SmeT-DNA is needed to further confirm this mechanisms. These *in vitro* data correlate with the *in vivo* induction of the expression of *smeDEF* in the presence of triclosan, indicating that the latter is a good inducer of the expression of the MDR efflux pump *smeDEF*. It might be predicted that this increased expression of *smeDEF* would lead to a reduction in the MIC similar to that observed in the strain *S. maltophilia* D457R, which harbors a defective allele of SmeT that cannot repress *smeDEF* expression. However, the increase in triclosan-induced resistance, although consistent, is modest (2.5-fold) in comparison to that observed for the *S. maltophilia* D457R mutant that overexpresses *smeDEF* (8-fold). Although the induction of *smeDEF* expression achieved by triclosan (8.7-fold) is slightly lower than that observed in the *S. maltophilia* D457R multidrug resistant mutant compared to its isogenic wild-type strain *S. maltophilia* D457 (13.7-fold), it is unlikely that these small differences can account for the differences in MICs observed when the expression of *smeDEF* is induced by triclosan or when it is constitutively de-repressed in the *S. maltophilia* D457R mutant. The small increase in the observed triclosan-induced resistance might be the consequence of the methods used in the assay. However, several compounds have a concentration-dependent effect on their target organism (beneficial at low concentrations and harmful at high concentrations), a behavior known as hormesis [70]. In the case of triclosan, the biocide induces expression of resistance determinants but simultaneously inhibits bacterial growth (Figure 8). In this case, the dual role described in hormesis occurs within too narrow a window of triclosan concentrations for both effects to be distinguished. This allows a working model to be proposed in which triclosan induces the transient over-expression of *smeDEF*, but the phenotypic consequences of this overexpression with respect to antibiotic resistance are counteracted by the inhibitory activity of the biocide itself (Figure 8). The observed increase in antibiotic resistance is therefore lower than might be predicted.

The resolution by X-ray diffraction of the structure of the SmeT-triclosan complex crystal allows more insight to be gained into the structure-function relationship of the SmeT transcriptional repressor. This paper is the first to describe the structure of triclosan bound to a transcriptional regulator and of SmeT complexed with a ligand. The SmeT-triclosan complex and the apo SmeT (PDB code: 2W53) structures are almost identical, with an rmsd value of 0.345 Å. However, in the present SmeT-triclosan complex structure nearly all the residues could be modeled while most of those in the N-terminal domain of subunit B were disordered in the apo SmeT structure, even though they were crystallized under the same conditions and show a similar crystal packing lattice (Figure 5). This observation supports the hypothesis that the N-terminal domain of SmeT is intrinsically disordered, being stabilized upon ligand or DNA binding. It is in the interface between the N-terminal domains of both subunits where this effect is most remarkable. The stabilization of this domain due to the binding of the effector reveals a new interface area between the loop connecting helices α6 and α7 and the top of helix α1 (Figure 6b). An analogous contribution to the dimer interface through the DNA binding domain has been described in the TtgR complex [23].

**Figure 9:**
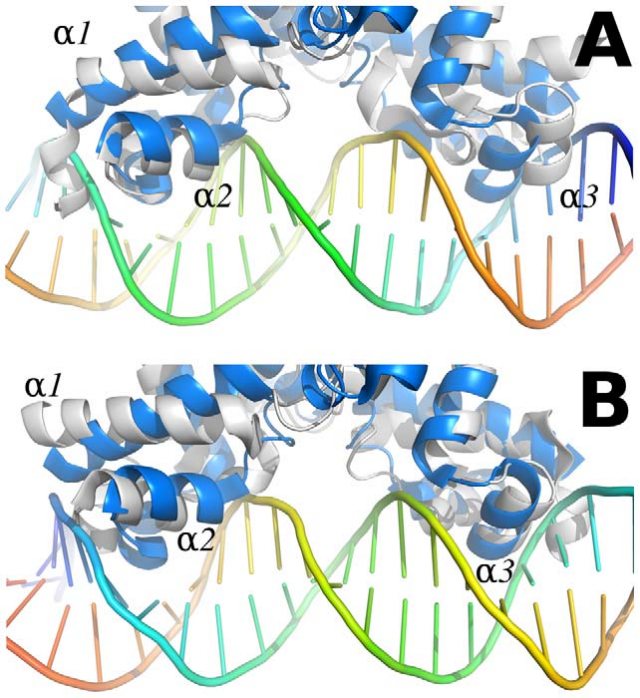
Comparison between the SmeT-Triclosan (in grey) and the QacR-DNA complex (in blue) structures. Chain A of the SmeT-Triclosan complex structure was superposed with QacR in the QacR-DNA complex structure (PDB entry 1JT0 [64]). The recognition helices α1 and α2 of this chain are placed in the same position than the corresponding helices of QacR (left side in panel A, right side in panel B). On the contrary, helix α1 in chain B is rotated, and helices α2 and α3 are displaced clashing against the DNA (right side in panel A, left side in panel B). This conformation prevents the repressor from binding to DNA. The views shown in panels A and B are obtained by a 180 degrees rotation perpendicular to the DNA axis.

Due to the stabilization of the SmeT structure, the distance between the α-3 recognition helices of both subunits could be measured in the SmeT-triclosan complex, with 44.6 Å between the C-alpha atoms of Tyr49 (the N terminal of subunit B in the apo SmeT structure had to be modeled in order to estimate this length). This value is 10 Å longer than the distance between the B-form DNA major grooves, where these helices should bind (Figure 9). This conformation prevents the repressor from binding to DNA, as observed for the QacR dimer [25]. Further, the conformational change associated with the binding of triclosan causes the release of SmeT from its DNA operator, as seen in the EMSA assay.

The present work shows that two molecules of triclosan enter the hydrophobic binding pocket of SmeT. The binding of two molecules to one subunit of a dimer has been reported for other member of the TetR family, e.g. the binding of two molecules of phloretin to TtgR [23] or the binding of two different drugs to QacR [64]. One molecule of triclosan binds at the bottom of the binding pocket, parallel to helix α6, in a geometry similar to the phloretin molecule when binding to the high affinity site of TtgR. The second molecule, binds close to the dimer interface, and also interacts with this helix through the phenolic ring (Figure 6b). The first molecule of triclosan stacks against the phenol ring of Phe70, a residue identified as the gatekeeper of the ligand binding pocket. Supporting this hypothesis, the so-called open conformation is the only one seen in the structure of the SmeT-triclosan complex. In contrast, Phe70 has a double conformation in the apo SmeT structure.

The ligand binding site of SmeT is mainly formed by hydrophobic residues - there are just three polar residues: His67, His167 and Ser96. This lack of charged residues may be related to the polyspecific substrate recognition of SmeT. Additionally the binding site can increase its volume almost 2-fold by displacing the side chains of residues with double conformations in the apo SmeT structure namely Phe70, Met113 and Leu114. The different side chains that can be exposed, or covered, may also facilitate interactions with a wide range of compounds. Remarkably, some residues displaced by ligand binding (Met110 and Met113) belong to helix α6, which interacts through hydrogen bonds with residues from helix α1. These interactions are described as decisive in QacR and TetR deactivation due to ligand binding [19]. Through these interaction networks between helices, the QacR and TetR changes the relative orientation of the N-terminal domains to generate a ligand bound structure unable to bind DNA. The present data suggest that SmeT may modify its shape after triclosan binding, provoking the release of DNA by a similar mechanism (Figure 9).

The structure of the complex SmeT-triclosan, together with the experimental data supporting its role in *smeDEF* pump derepression, is the first structural evidence that triclosan can act as an effector of an MDR system regulator, and provides an unambiguous link between the presence of this biocide and the increased efflux of antibiotics by the opportunistic pathogen *S. maltophilia*.

## Materials and Methods

### Protein purification and crystallization

SmeT protein was expressed and purified as previously described [53]. Briefly, the *smeT* gene was cloned into the pTYB1 vector (IMPACT-CN system, New England Biolabs) and the protein expressed in the *E. coli* strain ER2566 (IMPACT-CN system, NEB). After induction with 0.5 mM IPTG, the culture was grown overnight at 15°C. Cells were disrupted by sonication and centrifuged. The supernatant with the SmeT-Intein-CBD fusion protein was loaded into a Poly-Prep chromatography column (Bio-Rad) containing chitin beads (NEB). After overnight incubation in cleavage buffer (20 mM Tris-HCl, 0.5 M NaCl, 100 mM dithiothreitol (DTT), 1 mM EDTA, pH 8.0) at 4°C, SmeT was eluted and the remaining DTT present in the sample removed by dialysis against Tris-buffered saline. Finally, the sample was loaded into a Sephacryl S100 gel filtration column (GE Healthcare) in a buffer containing 300 mM NaCl and 20 mM Tris pH 8.0.

Prior to concentration, the sample was incubated with triclosan (5-chloro-2-(2,4-dichlorophenoxy) phenol), previously dissolved in DMSO, for 4 h at 4°C in a 1∶10 molar ratio. The SmeT-triclosan complex was concentrated to 5 mg/ml and crystallized using the sitting-drop vapor diffusion technique in a solution containing 175 mM Li_2_SO4, 100 mM Tris pH 8.5 and 27% v/v PEG MME 2K. Crystals grew to approximately 500×200×20 µm within 3 days at 22°C. These crystals were transferred into a cryoprotectant solution (175 mM Li_2_SO4, 100 mM Tris pH 8.5 and 30% v/v PEG MME 2K) and flash-cooled at 100°K.

### Crystallographic data collection and processing

Crystallographic data were collected at the Beamline ID23-2 of the European Synchrotron Radiation Facility (ESRF) and processed using Mosflm [71] and SCALA software [72]. The crystal parameters were equivalent to those of the apo SmeT structure [53]. The crystals belonged to the space group P2_1_ and had unit cell dimensions of a = 56.5, b = 59.5, c = 84.8 Å, and β = 102.7°. The Mathews coefficient for a dimer in the asymmetric unit was 2.82 Å^3^/Da, which corresponds to a solvent content of 56.4%. Details of data collection, crystal parameters and data-processing statistics are given in Table 1.

**Table 1:** Data collection, processing and refinement statistics.

Data collection	
Space group	P2_1_
Unit Cell Parameters (a, b, c (Å); β (°)	56.2, 59.3, 84.6; β = 102.9
Resolution (Å)	2,02
No. of measured reflections*	126,007 (18,214)
No. of unique reflections*	35,800 (5179)
Completeness (%)*	99.9 (100)
R_meas_ (%)*	10.3(57.2)
Multiplicity*	3.5(3.5)
I/σ(I)*	9.5 (2.8)

Refinement	
R_cryst_/R_free_ (%)	20/25
No. of atoms	
Protein	3356
Solvent	225
Ligand	34
B-factors (Å^2^)	
Protein	35
Solvent	34
Ligand	48
RMSDs	
Bond lengths (Å)	0.023
Bond angles (°)	1.85

*Values in parentheses are for the high resolution shell (2.13–2.02 Å).

### Model refinement and validation

Difference Fourier techniques were used to solve the structure since unit cell dimensions are almost identical to those of the SmeT structure (PDB code 2w53 [53]). Molecular Replacement using MOLREP [73] has confirmed that the crystal packing of the SmeT-TCL complex is identical to that of the SmeT protein. The translation-libration-screw (TLS) groups (3 per chain) were defined using the TLSMD server [74]. Iterative cycles of manual building and TLS-restrained refinement cycles were performed using COOT [75] and REFMAC software [76] to final Rfree and Rcryst values of 25 and 20% respectively (Table 1). The final model comprised residues 6 to 218 of chain A, 10 to 218 of chain B, 225 water molecules, 2 sulphate anions and 2 triclosan molecules. The overall electron density map was of high quality but of poor definition for residues 30–31 and 120–121 in chain B, perhaps due to the high flexibility of these regions. Analysis of the geometry for the final model was performed using Molprobity software [77]; 98.3% of the residues fell within the favored regions of the Ramachandran plot and none within the disallowed regions. The anisotropy of the atomic displacement parameters was analyzed using the PARVATI server [78]; the mean anisotropy for the dimer was 0.46±0.19 (0.43±0.19 for chain A and 0.48±0.19 for chain B). Protein surfaces were analyzed using the PISA server [79]. Cavity volumes were determined using Casp software [80]. Figures were produced using PyMOL software [81].

### Fluorescence measurements

The dissociation constant for SmeT was obtained from the change in the intrinsic fluorescence of the protein upon triclosan binding. Mixtures consisting of 0.5 µM SmeT with various concentrations (7–1000 nM) of triclosan were prepared in 50 mM NaCl, 20 mM Tris pH 8.0 and 5% (v/v) ethanol. Fluorescence emission spectra were recorded at room temperature in a QuantaMaster QM-2000-7 model spectrofluorometer (Photon Technology International) using a 1-cm-pathlength quartz cell (Hellma). The excitation monochromator was set at 280 nm and emission was recorded between 310 and 360 nm. Three independent titration curves were carried out. The data were analyzed using a non-linear least-squares fit assuming an stoichiometry of two molecules of triclosan per SmeT dimer.

### DNA labeling and EMSA with biocides

The γ-[^32^P] dATP labeling of the 30-bp oligonucleotide ^5′^ GTTTACAAACAAACAAGCATGTATGTATAT ^3′^ that contained the operator site, and the SmeT purification, were performed as described in earlier work [53]. EMSA assays with or without triclosan were performed by incubating the 5′ end-labeled 30 bp double stranded DNA (2 nM, 10000 cpm) with 0.2 µM SmeT for 20 min at room temperature. The binding buffer used was 10 mM Tris-HCl, 50 mM KCl, 10 mM MgCl_2_, 1 mM EDTA, pH 7.2, 50 µg/ml bovine serum albumin, 1 mM dithiothreitol, 5% (v/v) glycerol, and 100 µg/ml poly(dI-dC) as nonspecific competitor DNA. Increasing concentrations (0.1 mM and 0.2 mM) of the biocide were then added and incubated at room temperature for 15 min more. Retarded complexes were separated on a 6% non-denaturing polyacrylamide gel (40∶1 acrylamide∶bisacrylamide). The electrophoretic conditions were: 100 V for 90 min at 4°C. The buffer used was TE (89 mM Tris-borate, 2 mM EDTA pH 8.0). Gels were dried before autoradiography.

### RNA preparation and real time reverse transcription PCR

15 µl of overnight cultures of strains *S. maltophilia* D457 or *S. maltophilia* D457R were used to inoculate flasks containing 15 ml LB broth with or without sub-inhibitory concentrations of the biocide (15 µg/ml). When the OD_600_≈1.0, cells were spun down at 6000× g for 10 min at 4°C and immediately frozen on dry ice and stored at −80°C. Total RNA was extracted from the pellets using the RNeasy Mini Kit (QIAGEN) according to the manufacturer's instructions. TURBO DNA-free (Ambion) was used to eliminate any remaining DNA. RNA integrity was verified on a 1% agarose gel and the absence of DNA confirmed by real time PCR using *gyrA(+)*: ^5′^CCAGGGTAACTTCGGTTCGA^3′^ and *gyrA(−)*: ^5′^GCCTCGGTGTATCGCATTG^3′^ primers. cDNA was obtained from 1 µg RNA using the High Capacity cDNA Reverse Transcription Kit (AB Applied Biosystems). Real time RT-PCR was performed as described elsewhere [82]. Briefly, a first denaturation step was allowed at 95°C for 10 min followed by 40 cycles (95°C for 15 s, 60°C for 1 min) for amplification and quantification. Primers were designed using Primer Express 3.0 software (AB Applied Biosystems) with the default settings. *RT-smeC(+)*: ^5′^TCACTGGATGCCTCGAAGATT^3′^ and *RT-smeC(−)*: ^5′^CAGGGCATCGGCCACTT^3′^ amplify a 93 bp fragment of the *smeC* gene. *RT-smeD(+)*: ^5′^CGGTCAGCATCCTGATGGA^3′^ and *RT-smeD(−)*: ^5′^TCAACGCTGACTTCGGAGAACT^3′^ amplify a 76 bp fragment of the *smeD* gene. *RT-smeJ(+)*: ^5′^TCGAACGCGCCTGAGTATC^3′^ and *RT-smeJ(−)*: ^5′^CGCTTTCGTACTGTGCCACTT^3′^ amplify a 96bp fragment of the *smeJ* gene. *RT-smeY(+)*: ^5′^AGCTGCTGTTCTCCGGTATCA^3′^ and *RT-smeY(−)*: ^5′^CACCAGGATGCGCAGGAT^3′^ amplify a 65 bp fragment of the *smeY* gene. *RT-gyrA(+)* and *RT-gyrA(−)* were used to amplify a 60 bp fragment of the housekeeping gene *gyrA* [83]. Differences in the relative amounts of the mRNA for the *smeC*, *smeD*, *smeJ* and *smeY* genes were determined using the 2^−ΔΔCt^ method [84]. RNA samples were extracted in three different experiments; the results are the mean values.

### Determination of susceptibility to antibiotics and antagonism assays

A diluted *S. maltophilia* D457 overnight culture (3∶100,000) was poured onto agar Mueller Hinton plates. Twenty minutes later, ciprofloxacin Etest strips (AB BioMérieux) were added. For the antagonism assays a square of dried Whatman paper previously soaked with 30 µl of triclosan at a concentration of 1 mg/ml, or in ethanol (control), was placed just below the point of the Etest strip corresponding to the minimal inhibitory concentration (MIC) of ciprofloxacin. Bacteria were grown for 24 h at 37°C.

### Determination of the effect of triclosan, antibiotics and their combinations on the growth rate of S. maltophilia

Experiments were performed in microtitre 96-well plates. Briefly, a diluted (1∶1000) *S. maltophilia* D457 overnight culture was split into two aliquots; one was supplemented with triclosan to obtain a final concentration of 3 µg/ml, the other was used as control. 198 µl of bacteria, with or without triclosan, were loaded per well, and 2 µl of antibiotic were added. The final antibiotic concentrations were: nalidixic acid 6 µg/ml, ofloxacin 1 µg/ml, norfloxacin 6 µg/ml and ciprofloxacin 1 µg/ml. Bacteria were grown at 37°C for 30 h and the OD_595 nm_ measured every 20 min using a Tecan Infinite 200 plate reader. Growth curves were plotted using Microsoft Excel.

### Accession numbers

PDB accession code 3P9T. The sequence of the *smeT* gene and the *smeT-smeD* intergenic region is deposited at the EMBL nucleotide sequence database under accession number AJ316010. The nucleotide sequence of *smeDEF* is deposited at the EMBL database under accession number AJ252200.
